# Supplementary material for: DIAPH3 deficiency links microtubules to mitotic errors, defective neurogenesis, and brain dysfunction
Source: eLife. 2021 Apr 26;10:e61974. doi: 10.7554/eLife.61974 (PMC8102060; doi:10.7554/eLife.61974)
Supplement: Supplementary file 1. — Over 81% of DIAPH3 knockdown cells exhibited mitotic errors (4.2% in control cells). These errors were divided into five ‘non-exclusive’ categories: (1) defects in chromosome alignment (57.4%); (2) abnormal astral microtubule (MT) (52.2%); (3) abnormal number of centrosomes (40%); (4) abnormal mitotic spindle (53%); and (5) mis-localization of SPAG5 (60%). n = 118 cells transfected with scrambled shRNA and 115 with sh-DIAPH3 from five individual experiments. [file elife-61974-supp1.docx]

**Supplementary File 1**

|  | Scrambled-shRNA (%) | sh-DIAPH3 (%) |
| --- | --- | --- |
| Abnormal cell division | 4.2 | 81.7 |
| (1) defect in chromosome alignment: lagging  chromosomes and/or asymmetric metaphase plate | 4.2 | 57.4 |
| (2) abnormal astral microtubule | 0.0 | 52.2 |
| (3) abnormal number of centrosomes | 0.0 | 40.0 |
| (4) abnormal mitotic spindle | 0.0 | 53.0 |
| (5) mis-localization of SPAG5 | 0.0 | 60.0 |
