# Supplementary material for: DIAPH3 deficiency links microtubules to mitotic errors, defective neurogenesis, and brain dysfunction
Source: eLife. 2021 Apr 26;10:e61974. doi: 10.7554/eLife.61974 (PMC8102060; doi:10.7554/eLife.61974)
Supplement: Supplementary file 2. — n = 4 embryos for each genotype. Student’s t-test, *p<0.05, **p<0.01. [file elife-61974-supp2.docx]

**Supplementary File 2**

|  | | % change in protein expression (*Diaph3* cKO/Ctrl) | *P*-value |
| --- | --- | --- | --- |
| *Cell Polarity Proteins* | | | |
| NUMA | | -25.87 | 0.126 |
| GPSM2** | | -46.47 | 0.002** |
| INSC | | -28.51 | 0.129 |
| NUMB* | | -36.27 | 0.025* |
| PAR3* | | -41.7 | 0.025* |
| *Motor Proteins* | | | |
| Dynein | | -32.95 | 0.055 |
| Dynactin | | -21.13 | 0.056 |
| *MT plus-end binding Proteins* | | | |
| SPAG5* | | -37.14 | 0.040* |
| KNSTRN* | | -55.8 | 0.043* |
| CLASP1 | | -10.94 | 0.063 |
| *Centromere Protein* | | | |
| CENPA* | -52.29 | | 0.045* |
